# Injury-related mortality among adolescents: findings from a teaching hospital's post mortem data

**DOI:** 10.1186/1756-0500-3-124

**Published:** 2010-05-05

**Authors:** Sally-Ann Ohene, Yao Tettey, Robert Kumoji

**Affiliations:** 1 World Health Organization Country Office in Ghana, Accra, Ghana; 2 Department of Pathology, University of Ghana Medical School, Korle Bu, Accra, Ghana

## Abstract

**Background:**

Injuries are noted to be an important cause of death among adolescents. There is however limited data on the injury related deaths among adolescents in Ghana.

**Findings:**

Using data from post-mortem records derived from the Department of Pathology of the Korle-Bu Teaching Hospital (KBTH), Accra Ghana from 2001 to 2003, the causes of injury related deaths among adolescents 10 to 19 years were analyzed by gender and age groups 10 to 14 and 15 to 19 years. There were 151 injury-related deaths constituting 17% of the autopsies performed among adolescents in the study period. The male-to-female ratio was 2.1:1. Drowning was the most common cause of death (37%) in the study population. This was followed by road traffic accidents (RTA) (33%). Over 70% of the RTA victims were pedestrians knocked downed by a vehicle. Deaths from electrocution, poisoning, burns, stab/gunshot, hanging and other miscellaneous causes (example blast injury, traumatic injury from falling debris, fall from height) made up the remaining 30% of the injury related mortality. Among males and in both age categories, drowning was the leading cause of death. In females, the highest mortality was from road traffic accidents accounting for almost half (49%) of the deaths; significantly more than that occurring in males (25%, p = .004).

**Conclusions:**

Findings from Korle-Bu Teaching Hospital post-mortem data on adolescents show that drowning and road traffic accidents are the leading causes of injury-related mortality. Appropriate injury reducing interventions are needed to facilitate a decrease in these preventable deaths.

## Background

One of the major threats to adolescent health is injury [1-6]. Apart from the significance of being a leading cause of death in young people, injuries are also recognized as a major contributory factor to morbidity, disability and healthcare and other costs such as lost future work and quality of life [4,7]. In industrialized countries injury related mortality from accidents, murder and suicide contribute to over 75% of deaths among adolescents and young adults [5]. After HIV/AIDS and other infectious diseases, injuries both intentional and accidental are estimated to contribute to one of four deaths among 15 to 29 year olds in Africa [3].

Previous reports show that among unintentional injuries, fatalities from road traffic accidents (RTA) are quite prevalent [8,9]. Other causes of injuries leading to death among adolescents cited in the literature are drowning, hanging, firearms and stab wounds and burns. Much of these study findings however come from other countries as there is limited injury related mortality data among adolescents in African countries in general and Ghana in particular [8-12]. The paucity of injury related mortality data and in fact data on causes of death in adolescents can be traced in part to the limited registration of deaths in several developing countries [13]. In Ghana this is reported to be less than 25% [13].

To have an understanding of injury related mortality among young people in Ghana, and in the absence of reliable death registration data, the study objective is to examine the prevalence and distribution of deaths due to injury among adolescents 10 to 19 years using available post mortem data from Korle Bu Teaching Hospital, Accra. It is expected that the information obtained would throw more light on injury related mortality among adolescents and inform efforts aimed at reducing these preventable deaths.

## Methods

Post mortem records of all adolescents 10 to 19 years performed at the Korle Bu Teaching Hospital (KBTH) in Accra, Ghana from the beginning of January 2001 to the end of December 2003, were retrieved from the autopsy logbooks of the Department of Pathology for this descriptive retrospective study. All cases with complete data on the date the post mortem was performed, the age, gender, referral source of the autopsy request, the cause of death/underlying disease and the cause of death coded according to the International Classification of Diseases (10^th ^Edition ICD, World Health Organization) [14] were entered into Microsoft Excel Sheet. All cases coded to reflect death related to injuries both intentional and unintentional were selected to constitute the sample for this study. The data was imported into STATA Data Analysis and Statistical Software version 9 for the analysis. Stata, (Version 9, College Station, TX)

For the analysis, the cases were grouped into two age groups, young and older adolescents, 10 to 14 years and 15 to 19 years respectively. The frequencies and proportions of the various injuries leading to death among the study population were determined and the cause of death by age group and gender were described. Chi-square tests were used to test for significant differences in cause of death between males and females and the two age groups with p < .05 taken as the level of significance. Ethical clearance for the study was given by the Ethical and Protocol Review Committee of the University of Ghana Medical School.

## Results

Over the period from January 2001 to December 2003, a total of 14,034 autopsies were conducted in the Department of Pathology with nine hundred and twenty seven (7%) being performed on adolescents 10 to 19 years. There were 882 adolescent autopsies with complete records over the three year period. Out of this number, 151 representing 17%, were injury related deaths and all were coroner's cases. In this sample, 102 were males (68%) and 49 females (32%) giving a male to female ratio of 2.1:1. The mean age of the males was 16.0 years while that of the females was 14.8 years. The older adolescents 15 to 19 years numbered 101 (67% of the study population). About half of the young adolescents (26) were boys, while among the older adolescents; three quarters of them were male.

**Figure 1:**
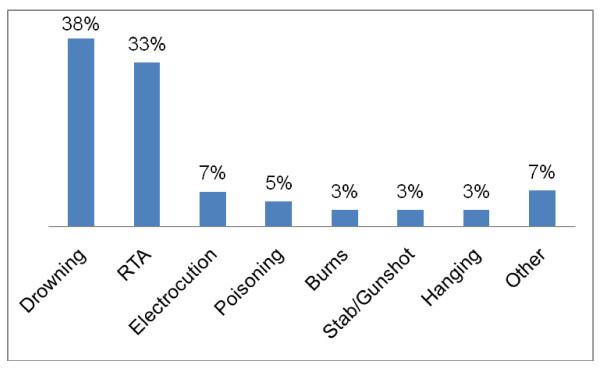
**Cause of injury related deaths among adolescents 10 to 19 years**.

Drowning was the most common cause of death (38%) in the study population. This was followed by road traffic accidents which accounted for a third (33%) of the deaths. Among the 50 adolescents who died from RTA, over 70% of the victims were pedestrians knocked downed by a vehicle and 18% were passengers. Deaths from electrocution, poisoning, burns, stab/gunshot, hanging and other miscellaneous causes (example blast injury, traumatic injury from falling debris, fall from height) made up the remainder of the injury related mortality (Figure 1).

Among the young adolescents, almost half (46%) of the deaths were due to drowning while a third (34%) died as result of RTA (Table 1). One out of three deaths (33%) in the older adolescents was from drowning and about the same proportion died from RTA. Out of the total eleven deaths from electrocution, 7 occurred among the older adolescents. Poisoning accounted for seven of the deaths with six of them being among older adolescents. All the five deaths from hanging occurred among the older adolescents.

**Table 1:** Cause of injury related deaths among Ghanaian adolescents 10 to 19 years by gender and age group

				Young Adolescents	Older Adolescents	
	Female	Male		10-14 years	15-19 years	
Cause of Death	Number (%)	Number (%)	p-value	Number (%)	Number (%)	p-value
Drowning	12(25)	45(44)	0.020	23(46)	34(33)	0.141
Road Traffic Accident	24(49)	26(25)	0.004	17(34)	33(33)	0.870
Electrocution	4(8)	7(7)	0.748*	4(8)	7(7)	1.000*
Poisoning	4(8)	3(3)	0.215*	1(2)	6(6)	0.426*
Hanging	1(2)	4(4)	1.000*	0(-)	5(5)	0.171*
Burns	2(4)	3(3)	0.660*	1(2)	4(4)	1.000*
Stab/Gunshot Wound	0(-)	5(5)	0.175*	1(2)	4(4)	1.000*
Other	2(4)	9(9)	0.505*	3(6)	8(8)	1.000*

Total	49(100)	102(100)		50(100)	101(100)	

* Fisher's exact test

In females, the highest mortality was from road traffic accidents accounting for almost half (49%) of the deaths. One out of four (25%) female deaths was from drowning. On the other hand, among all the males, drowning was the most common cause of death (44%) followed by RTA (25%). Out of the 7 deaths from poisoning, 4 occurred in females. Among the five who died from hanging one was a female. All the stab/gunshot wounds leading to death occurred in males. There were 11 deaths from other miscellaneous injuries (namely blast injury, traumatic injury from falling debris, fall from height) and 9 out of these were males. In the bivariate analysis with the exception of there being strong evidence of more females dying from RTA compared to males (p = .004, χ^2 ^= 8.246) and more males dying from drowning compared to female (p = 0.02, χ^2 ^= 5.426, there were no other differences of statistical significance comparing males and females and the two age groups.

## Discussion

This study describes the causes of injury related mortality among adolescents using post-mortem data from a teaching hospital in Accra. The leading cause of death was found to be drowning followed by road traffic accidents. As reported in other studies, disaggregation by gender and age showed that males and older adolescents were more affected by fatal injuries than their female and younger counterparts [4,5,8,9]. Among females the predominant cause of death from injuries was RTA.

The 17% injury mortality rate is relatively higher than the 9% deaths from injury among 10 to 30 year olds reported by Mock using hospital data from a district hospital in Ghana [15]. Using mortuary statistics from a teaching hospital in another city in Ghana, London and colleagues also reported 11.3% injury-related deaths among 15 to 44 year olds [16]. It is possible that the lower figures in these studies may be attributed to the differences in the age ranges and localities being compared. On the other hand even though our figure is nowhere near the high rates cited in the literature from western countries, there is still room for concern considering that injuries are a preventable cause of death [3,5,17].

Unlike other studies in which RTA is cited as the leading cause of injury death among adolescents [4,5,8,9,17], surprisingly, drowning was found to be the most common cause of fatal injuries among the Ghanaian adolescents studied. Among 10 to 14 year old adolescents in Mexico, drowning ranked second to motor vehicle traffic as a cause of injury death while among the older adolescents, it ranked fourth [9]. Drowning also ranked fourth as the cause of injury-related deaths among adolescents in India [8]. The data source used for this study does not allow for disaggregation into the type of water body in which the drowning occurred nor the circumstances leading to the drowning. With the study population drawn from Accra, a coastal town, it might be assumed that a considerable number of the drowning incidents occurred in the sea especially as public holidays are popular occasions for going to the beach in Accra. There is however a need to investigate this further as in a study by Shetty on the epidemiology of drowning in a coastal area in India, more deaths occurred in wells/ponds and rivers than in the sea [18]. Another angle for further exploration is the role of alcohol in these deaths from drowning [1]. In a study by Wintemute et al, alcohol use was a major risk factor for drowning among males 15 to 19 years with 38% of the drowning being alcohol associated [19]. However, regardless of where or how the drowning occurs, there is a need to strengthen preventive measures including supervision by life guards near water bodies/swimming areas and making available a rapid emergency response system that includes personnel skilled in resuscitation. Pan and colleagues noted a declining trend in injury-related mortality among Canadian adolescents including deaths from drowning (which ranked fifth) and suggested this may be due to an improvement and expansion in a number of passive preventive measures including the availability and quick response of paramedic services [4]. There is also the need for education on the danger of mixing alcohol and water-oriented recreation and measures to prevent the use of alcohol around water activities [1,19].

Even though RTA was not found to be the leading cause of deaths in our study, the proportion of adolescents dying from RTA was comparable to what has been reported [1,8]. In keeping with the literature, our study showed RTA to be the most common cause of injury deaths among females [4,5,12]. While RTA was not found to be the predominant cause of death for males as reported by other researchers [4,8,12], particularly among older adolescents it was equally as important as drowning for causing fatality in this study. A high proportion of the RTA victims in our study were pedestrians. This is not uncommon in less developed countries where the increasing number of vehicles, poor vehicle and road maintenance and low enforcement of traffic safety regulations in developing countries put vulnerable road users at risk for traffic fatalities [1-3]. Street vending in which young people weave in and out of moving traffic selling merchandise may also be a contributing factor. Reduction in mortality from traffic accidents will require educating young people on road safety and enforcing legislation on traffic laws and road safety regulations [1].

The other causes of death though contributing to lower proportions of mortality are also a cause for concern. Electrocution for example is not a commonly cited cause of death among adolescents and raises the question as to antecedents for this means of fatality in our study victims. Almost all the victims of poisoning, burns, hanging, gunshot wounds and miscellaneous causes of death were older adolescents with males being more affected in consonance with other reports [1]. The 3.3% death from hanging is close to that found among adolescents in India (4%) [8] but relatively lower than the 9.9% reported in a Canadian study by Sauvageau [12]. If it is to be inferred that those who died from hanging may have committed suicide then death from suicide in our study is lower compared to what is found in other studies [4,12,17]. However this interpretation may be flawed as some of the deaths from poisoning and even drowning could have been secondary to suicidal intent though this is not verifiable from the data set [8,18]. This calls for further investigation using a wider scope of data sources to be able to allow for analysis into the mechanism and circumstances of the deaths.

The limitations of this study include the use of post-mortem data from a teaching hospital in Accra which may not be nationally representative and the relatively small sample size. It must be emphasized therefore that the findings cannot be generalized to all adolescents in Ghana.

## Conclusions

This study throws some light on injury-related deaths among adolescents providing information that could guide prevention efforts. The study showed that drowning and road traffic accidents were responsible for the most deaths from injury in adolescents. To effectively address these preventable causes of death, much attention should be paid to instituting preventive measures including aquatic safety education, ensuring supervision near recreational water bodies and instituting and enforcing regulations that make our roads safe.

## List of abbreviations

HIV/AIDS: Human Immunodeficiency Virus/Acquired Immuno-Deficiency Syndrome; RTA: Road traffic accidents.

## Competing interests

The authors declare that they have no competing interests.

## Authors' contributions

YT and SAO conceptualized the study. YT and RK compiled and entered the data and SAO drafted the manuscript including the analysis. All authors read, edited and approved the final manuscript.
